# DNA Methylation in the Malignant Transformation of Meningiomas

**DOI:** 10.1371/journal.pone.0054114

**Published:** 2013-01-22

**Authors:** Fan Gao, Lingling Shi, Jonathan Russin, Liyun Zeng, Xiao Chang, Shuhan He, Thomas C. Chen, Steven L. Giannotta, Daniel J. Weisenberger, Gabriel Zada, William J. Mack, Kai Wang

**Affiliations:** 1 Zilkha Neurogenetic Institute, Keck School of Medicine, University of Southern California, Los Angeles, United States of America; 2 Department of Neurosurgery, Keck School of Medicine, University of Southern California, Los Angeles, United States of America; 3 USC Epigenome Center, Keck School of Medicine, University of Southern California, Los Angeles, United States of America; 4 Department of Psychiatry, Keck School of Medicine, University of Southern California, Los Angeles, United States of America; 5 Division of Bioinformatics, Department of Preventive Medicine, Keck School of Medicine, University of Southern California, Los Angeles, United States of America; University of Illinois College of Medicine, United States of America

## Abstract

Meningiomas are central nervous system tumors that originate from the meningeal coverings of the brain and spinal cord. Most meningiomas are pathologically benign or atypical, but 3–5% display malignant features. Despite previous studies on benign and atypical meningiomas, the key molecular pathways involved in malignant transformation remain to be determined, as does the extent of epigenetic alteration in malignant meningiomas. In this study, we explored the landscape of DNA methylation in ten benign, five atypical and four malignant meningiomas. Compared to the benign tumors, the atypical and malignant meningiomas demonstrate increased global DNA hypomethylation. Clustering analysis readily separates malignant from atypical and benign tumors, implicating that DNA methylation patterns may serve as diagnostic biomarkers for malignancy. Genes with hypermethylated CpG islands in malignant meningiomas (such as *HOXA6* and *HOXA9*) tend to coincide with the binding sites of polycomb repressive complexes (PRC) in early developmental stages. Most genes with hypermethylated CpG islands at promoters are suppressed in malignant and benign meningiomas, suggesting the switching of gene silencing machinery from PRC binding to DNA methylation in malignant meningiomas. One exception is the *MAL2* gene that is highly expressed in benign group and silenced in malignant group, representing *de novo* gene silencing induced by DNA methylation. In summary, our results suggest that malignant meningiomas have distinct DNA methylation patterns compared to their benign and atypical counterparts, and that the differentially methylated genes may serve as diagnostic biomarkers or candidate causal genes for malignant transformation.

## Introduction

Meningiomas are central nervous system tumors that originate from the meningeal coverings of the brain and spinal cord. They are the most frequently diagnosed primary brain tumor, accounting for 33.8% of all primary brain tumors in the United States, with an annual incidence of 40 to 60 cases per million persons [1], [2]. Meningiomas are classified as benign (grade I), atypical (grade II), or anaplastic/malignant (grade III) according to the World Health Organization (WHO) histological grading criteria. They represent 80%, 15% and 5% of all meningiomas respectively [3]. Malignant meningiomas are typically associated with brain invasion, early recurrence, and decreased progression-free and overall survival [4], [5]. Surgical resection is the standard treatment for most symptomatic meningiomas, whereas malignant meningiomas are characterized by frequent recurrence typically mandating multimodality treatment consisting of repeated surgery, postsurgical radiation, and occasionally chemotherapy. Because the prognosis for malignant meningiomas remains much worse than benign or atypical meningiomas, any characterization of genetic or epigenetic processes involved in malignant transformation may provide important insights into novel diagnostic and therapeutic strategies aimed at understanding this aggressive tumor subtype.

Several genetic studies have been conducted over the past a few years to investigate the genetic basis of meningioma pathogenesis. A genome-wide association study including 859 meningioma patients and 704 control subjects identified the *MLLT10* gene as strongly associated with susceptibility to meningiomas [6]. Additionally, several studies have investigated meningioma tissue to identify somatic mutations that may be related to tumor progression. One recent study assessed copy number variation and gene expression patterns associated with meningioma samples of different grades to explore the genomic landscape of meningiomas [7]. The authors found that five categories of meningiomas, based on expression patterns, were generally correlated with histological grade, recurrent status and copy number variations, although tumor grades were not precisely tracked by expression pattern. Most of these studies, however, were not specifically targeted toward the malignant subtype of meningioma, which accounted for only a minor subset of all enrolled patients.

In addition to alteration in the DNA sequences, epigenetic modifications are also closely linked to cancer genesis and progression. Aberrant DNA methylation is one of the major types of epigenetic modifications in cancer [8]. DNA methylation in mammalian cells occurs by adding a methyl group to cytosine to generate 5-methylcytosine [9], [10], [11], mainly at cytosine-guanosine dinucleotides (CpGs). CpG–rich regions are called CpG islands, and are associated with all known housekeeping and some tissue-specific genes [12], [13]. It has been reported that global DNA hypomethylation is directly associated with tumor formation [14], [15], [16], [17], [18], [19]. Additionally, focal DNA hypermethylation at regional hotspots (including CpG islands) was observed in different human tumors [20], [21], [22], [23]. DNA methylation levels of *HOXA7*, *HOXA9* and *HOXA10* were reported significantly higher in WHO grade II and III meningiomas [24] than in grade I (benign) meningiomas. DNA methylation of a group of 6,157 genes was previously studied to identify biomarkers correlated with a high frequency of tumor recurrence in WHO grade I and II meningiomas [25]. However, variations in genome-wide DNA methylation have not been studied in malignant meningiomas, and it remains unclear how benign and malignant meningiomas vary with respect to epigenetic alterations.

In the current study, we use meningiomas as a model system to investigate the role of DNA methylation in tumor malignancy. To our knowledge, this is the first study to explore genome-scale DNA methylation in malignant, atypical and benign meningiomas. Additionally, unlike most previous cancer genetics studies that compare DNA methylation patterns between tumor and normal tissue, our goal was to investigate whether benign and malignant tumors differ in DNA methylation patterns, and if these differences have biological and clinical significance. We assayed genome-scale DNA methylation and mRNA expression levels of meningiomas categorized as malignant, atypical and benign according to the WHO criteria. Our analysis provides a mechanistic view to uncover the regulatory role of DNA methylation in meningioma malignancy. We identified a list of differentially methylated genes that could serve as diagnostic biomarkers or as candidate genes for further investigation.

## Materials and Methods

### Patients and tissues

The study was approved by the Institutional Review Board at the University of Southern California. Informed written consent was obtained for all participants (either from patients directly or a surrogate with the highest priority for decision making if the patient is unable to provide consent) prior to undergoing surgical tumor resection. All patients presenting to Los Angeles County – USC Medical Center or Keck Hospital of USC with benign or malignant neoplasms of the brain or spine were eligible participants. Demographic information was obtained from patient medical records. Tumor samples were procured by the clinical pathology service at the time of surgery. Specimens were brought directly to the pathology laboratory from the operating room for rapid processing. After sufficient tissue was utilized for diagnostic purposes and released by pathology, the remaining tissue was stored in sterile saline and snap frozen in a −80°C freezer.

### DNA methylation array

Genomic DNA samples were prepared by Qiagen DNeasy Blood & Tissue Kit using the manufacturer's recommended protocol. Genomic DNA was successfully extracted from all except one of the tissue samples collected. Genomic DNA extracted from the excluded sample had a very low DNA concentration and didn't pass the quality control (QC) test for the DNA methylation array. This sample was retrieved from the USC tissue bank, and no additional tissue sample was available for this subject. Genome-scale DNA methylation profiles of 482,421 methylation sites were assessed using the Illumina Infinium HumanMethylation450 (HM450) BeadChips. The array has 99% coverage of RefSeq genes, at gene promoter, 5′UTR, first exon, gene body, and 3′UTR. For DNA methylation data obtained after background correction, data points with a detection *P*-value >0.05 were replaced with “NA” values. Probes with one or more “NA” points were excluded. In addition, probes targeting the X or Y chromosomes were removed from the comparative analysis. The data was deposited into GEO with accession number GSE42882.

### Gene expression array

Total RNA samples were extracted from meningiomas tissue by Qiagen RNeasy Plus Universal Kit following manufacture's recommended protocol. The liquid nitrogen grinding method was used to homogenize tissue samples. Tissue samples were first cut into small fragments with no more than 100 mg material in a 1.5 mL tube. Liquid nitrogen was then added into the tube, and the tissue was grinded with grinding rod, before adding 900 µL QIAzol Lysis Reagent. The tubes were vortexed for 60 sec and left at room temperature for 5 minutes before following the standard RNeasy Plus protocol for RNA extraction. The quality of total RNA was assessed using Experion™ RNA StdSens Chip on a Bio-Rad Experion system (bioanalyzer). Genome-scale gene expression profiles of approximately 47,000 transcripts were quantified using an Illumina HumanHT-12 v4 Expression Beadchip. Raw data were processed and normalized without background subtraction using the Illumina GenomeStudio software suite.

### Data analysis

Clustering analysis was performed using MultiExperiment Viewer (MeV) software suite v.4.7 [26] with a loaded dataset of β values for the probes that are within top 2% of highest standard deviation across all meningioma samples. Two-way clustering was performed using Pearson's correlation and average linkage for both the sample tree and the gene tree. Differential DNA methylation based on β values was assessed using Significance Analysis of Microarrays (SAM) analysis with 100 permutations to identify significant genes, using two-class unpaired comparison between malignant and benign meningioma groups. The same method was also applied to evaluate gene expression data.

In the benign group, TSS200 DNA methylation probes having mean β values between 0.80 and 1 (6,834 probes) were selected as genes with heavily methylated promoters. To further identify genes significantly hypomethylated within this pool, differential mean β values between benign and malignant groups for each selected probe were calculated. The probes with differential mean β values greater than 0.6 were chosen for analysis.

Differential mean β values calculated for all the probes (genomic locations) together with the ChIP-Seq signals of EZH2, RING1B and H3K27me3 in human embryonic stem cells (GSE13084) were visualized using Integrative Genomics Viewer (IGV) [27]. Enrichment of pathway analysis was done using Gene Set Enrichment Analysis (GSEA) software suite [28]. Additional statistical analysis was performed using custom scripts in R software suite (http://www.r-project.org/).

Publicly available gene expression data for meningiomas were downloaded from Gene Expression Omnibus database (GSE16153), including expression array data for forty-three benign and six malignant tumor samples. Both DNA methylation and expression data collected from 27 brain glioma samples were downloaded from the TCGA Data Portal (https://tcga-data.nci.nih.gov/tcga/).

## Results

### Malignant meningiomas are globally hypomethylated compared to benign ones

**Figure 1:**
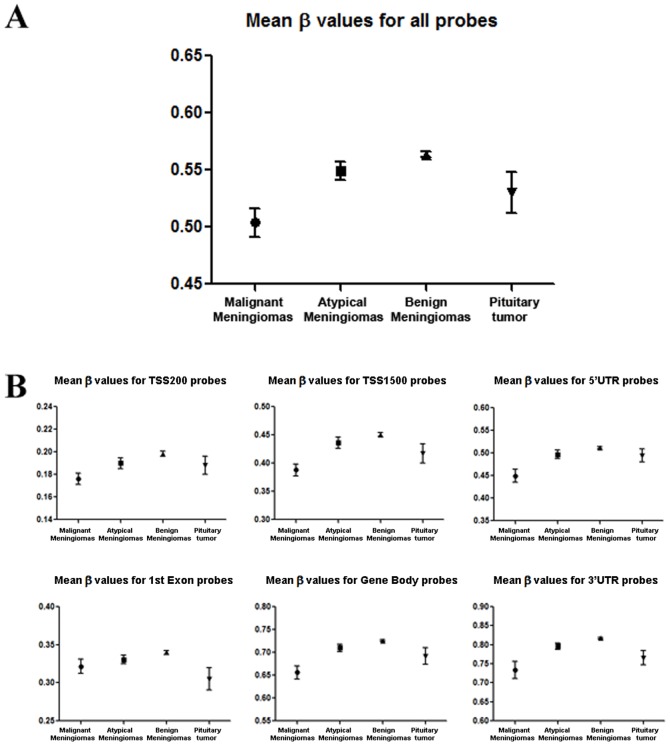
DNA methylation levels in different groups. Analysis of sample DNA methylation mean β values for all probes, TSS200 probes, TSS1500 probes, 1^st^ Exon probes, Gene Body probes, 3′UTR probes, and 5′UTR probes. Atypical and malignant meningiomas tend to have decreased levels of global DNA methylation in comparison to benign meningiomas.

Our study assayed DNA methylation in 19 primary brain tumor samples (ten benign, five atypical and four malignant meningiomas) from patients who underwent surgical tumor resection at the Keck Hospital of USC and the Los Angeles County – USC Medical Center. Patient characteristics are provided in Table 1. Following DNA extraction, genome-scale DNA methylation profiles were measured using the Illumina HumanMethylation450 Beadchip (HM450), which contains 482,421 probes across the genome. For comparative purposes, we also included four pituitary adenomas to serve as a control to assess the levels of DNA methylation across tumor types. The DNA methylation level for each probe is described as a β value, which is calculated as 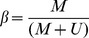, in which M and U represent the mean methylated and unmethylated signal intensities, respectively. The probes can be further classified as TSS200 (within 200 bp from transcription start sites), TSS1500, 1^st^ Exon, Gene Body, 3′UTR, and 5′UTR based on their relative location with respect to genes. We found that DNA methylation levels in malignant meningioma are significantly lower than those in atypical or benign tumors (Figure 1, *p*<0.05 by two-tailed t-test). Additionally, the DNA methylation levels of malignant meningiomas are, in general, lower than pituitary adenomas, although this result was not statistically significant (*p* = 0.269, two-tailed t-test). DNA methylation levels of benign meningiomas, however, are significantly higher than those of pituitary adenomas (*p*<0.05, two-tailed t-test). Atypical meningiomas also showed trends of DNA hypomethylation compared to benign tumors, which may reflect initiation of DNA hypomethylation (*p*<0.05, two-tailed t-test). Further analysis showed that probes targeting different gene regions such as TSS200, TSS1500, 1^st^ Exon, Gene Body, 3′UTR, 5′UTR all have decreased mean β values in malignant meningiomas, suggesting that DNA hypomethylation is not limited to specific gene regions but rather occurs throughout the genome. Therefore, global DNA hypomethylation is associated with malignant transformation of meningiomas as observed in atypical meningiomas. Altered DNA methylation levels may be associated with gene dysregulation.

**Table 1:** Demographics of Patients Harboring Meningiomas.

Patient	Sample ID	Age	Hospital	Gender	Race	Location	Chemo/Radiation	WHOGrade	Methylation	Gene expression
1	249	56	LAC	M	H	LEFT FRONTAL	NONE	III	Y	Y
2	362	68	LAC	F	A	LEFT FRONTAL	NONE	II	Y	N
2	489	44	LAC	F	H	OLFACTORY GROOVE	NONE	II	Y	N
4	479	60	LAC	F	H	LEFT SPHENOID WING	NONE	I	Y	Y
5	347	51	LAC	F	H	LEFT POSTERIOR CLINOID	NONE	I	Y	Y
6	392	53	LAC	F	H	LEFT SPHENOID WING	NONE	I	Y	Y
7	403	57	LAC	F	C	LEFT TEMPORAL	NONE	I	Y	N
8	010	58	KH	M	C	RIGHT OCCIPITAL	NONE	III	Y	Y
9	254	47	KH	F	C	LEFT PARIETAL, RIGHT PARIETAL	NONE	III	Y	Y
10	678	68	KH	F	H	LEFT FRONTAL/TEMPORAL	NONE	II	Y	N
11	238	61	KH	F	H	LEFT FRONTAL	NONE	I	Y	N
12	048	63	KH	F	C	RIGHT TEMPORAL	NONE	I	Y	Y
13	404	48	KH	F	C	LEFT FRONTAL	NONE	I	Y	Y
14	255	32	KH	F	A	OLFACTORY GROOVE	Chemo/Radiation	III	Y	Y
15	176	50	LAC	F	H	LEFT TEMPORAL	NONE	1	Y	N
16	221	38	LAC	M	H	LEFT FRONTAL	NONE	III	N	Y
17	274	35	LAC	M	H	LEFT PETROUS	NONE	I	Y	N
18	311	50	LAC	F	A	LEFT PARIETAL	NONE	I	Y	N
19	351	24	LAC	M	C	RIGHT FRONTAL	NONE	II	Y	N
20	320	55	LAC	M	H	LEFT FRONTAL	NONE	II	Y	N

Sample ID: Refers to sample number for analysis.

Age: Age at the time of surgery when the sample was obtained.

Hospital: LAC = LA County; KH = Keck Hospital of USC.

Race: C = Caucasian; H = Hispanic, A = Asian.

Chemo/Radiation: Refers to whether the patient received chemotherapy or radiation prior to surgery to obtain the specimen.

Methylation: Included in methylation analysis.

Gene Expression: Included in gene expression analysis.

### Hierarchical clustering reveals subtype-specific DNA methylation profiles in meningiomas

**Figure 2:**
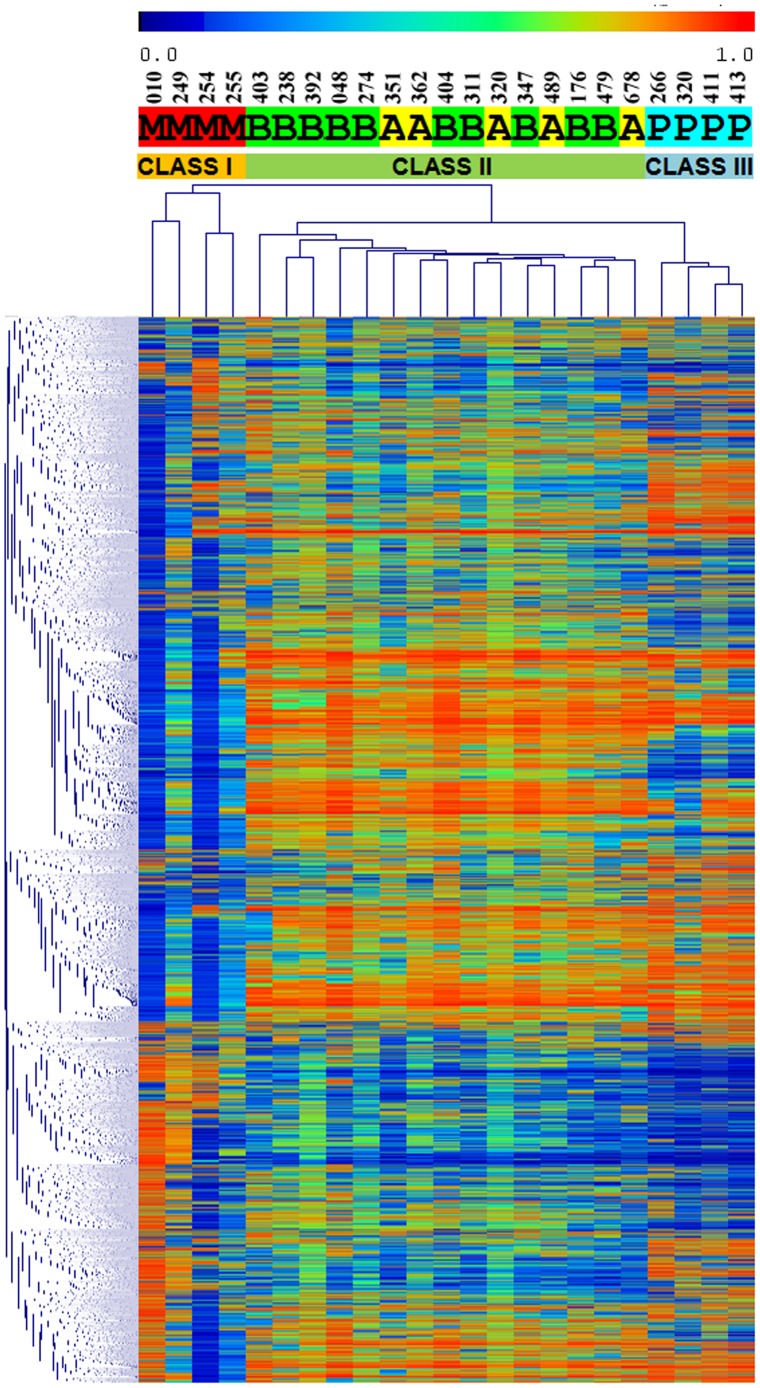
Hierarchical clustering based on DNA methylation. Malignant, atypical, benign meningiomas and pituitary adenoma samples were labeled with M, A, B and P, respectively. Malignant meningiomas can be readily separated from benign/atypical meningiomas or pituitary adenomas, but benign and atypical meningiomas are not separable based on methylation patterns alone.

To investigate whether global DNA methylation patterns can be predictive of WHO tumor grades, we performed hierarchical clustering analysis, using the top 2% of probes with highest variability (standard deviation) of DNA methylation levels across 19 meningiomas. Additionally, we included 4 pituitary adenoma samples in the same analysis to assess any similarity with different subtypes of meningiomas. Hierarchical clustering readily divides these samples into three distinct subgroups (Figure 2): Four samples in class I are malignant meningiomas, fifteen samples in class II are mixed benign and atypical meningiomas samples, and four samples in class III are pituitary adenomas. We noted that there is no clear separation of atypical meningioma samples from benign ones, despite the decreased global DNA methylation levels in the atypical samples. Furthermore, we noted that benign/atypical meningiomas actually cluster closer to pituitary adenoma than malignant meningiomas, suggesting the presence of drastic changes in global DNA methylation in malignant meningiomas. The heatmap shown in Figure 2 also indicates that most genomic regions selected in this analysis are hypomethylated in malignant meningiomas, similar to what we observed using all DNA methylation probes on the array. Therefore, DNA methylation patterns are correlated with malignancy of meningiomas, and may potentially serve as diagnostic biomarkers for malignancy.

### Biological pathway analysis for differentially methylated genes

To investigate whether genes affected by alterations of DNA methylation in malignant meningiomas tend to fall within specific molecular pathways, we next performed pathway analysis using the Gene Set Enrichment Analysis (GSEA) software suite [28]. This software evaluates whether an *a priori* defined gene set (pathway) is statistically different between two biological states. In our study, only probes targeting to gene core promoter regions (TSS 200 probes, total 58,141) were included in the analysis. When comparing the malignant *vs.* benign meningioma tumors, among all 639 genes sets defined in canonical pathways version 2.5 in the Molecular Signature Database (MSigDB), none of them are significantly enriched in the malignant group. In comparison, ten gene sets are enriched in the benign meningioma phenotype (higher β values) with FDR<0.25 and nominal *P* value <0.01 (Table 2). It is interesting to note that the glioma pathway is the top pathway enriched in the benign meningioma phenotype (Figure S1), suggesting the possibility that malignant meningiomas and gliomas may share some common molecular pathways toward malignant transformation.

**Table 2:** Identified pathways with differentially methylated gene promoters in malignant meningiomas.*

Rank	Gene sets in MSigDB	Size	ES	NES	NOM p-val	FDR q-val	FWER p-val
1	HSA05214_GLIOMA	32	−0.51	−1.79	0.003	0.119	0.072
2	HSA00561_GLYCEROLIPID_METABOLISM	30	−0.58	−1.74	0.004	0.141	0.16
3	HSA04540_GAP_JUNCTION	53	−0.55	−1.74	0.000	0.095	0.161
4	GLUCONEOGENESIS	25	−0.56	−1.72	0.006	0.080	0.177
5	GLYCOLYSIS	25	−0.56	−1.72	0.006	0.064	0.177
6	HSA00010_GLYCOLYSIS_AND_GLUCONEOGENESIS	33	−0.56	−1.72	0.002	0.058	0.189
7	GLYCEROLIPID_METABOLISM	21	−0.65	−1.70	0.004	0.058	0.218
8	HSA04060_CYTOKINE_CYTOKINE_RECEPTOR_INTERACTION	121	−0.52	−1.68	0.000	0.070	0.278
9	HSA04020_CALCIUM_SIGNALING_PATHWAY	74	−0.51	−1.64	0.000	0.093	0.383
10	ARGININE_AND_PROLINE_METABOLISM	20	−0.60	−1.56	0.009	0.185	0.611

* Analysis was performed to compare malignant and benign sample groups. Size – number of genes in a set; ES – enrichment score, represents the degree to which a gene set is overrepresented at the extreme of the ranked list; NES – normalized enrichment score, represents the corrected ES based on the size of the set; NOM p-val – nominal *P* value, represents statistical significance of the ES; FDR q-val – false discovery rate q value, represents statistical significance of false positive of a given NES; FWER p-val – familywise-error rate *P* value, represents statistical significance of a conservative correction to exclude any false-positive gene set.

### Relationship between DNA methylation and gene expression in primary meningiomas

**Figure 3:**
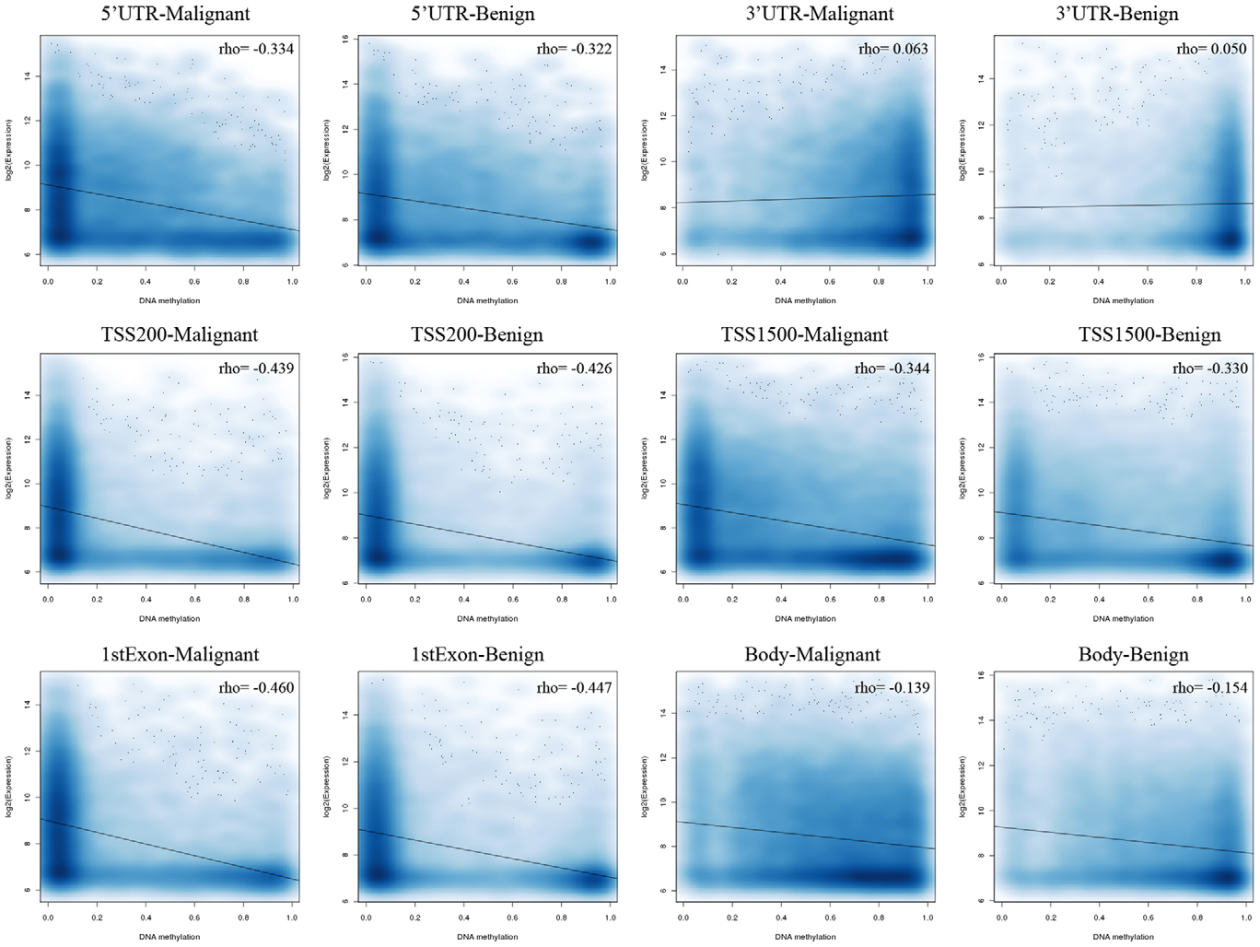
The relationship of DNA methylation and expression. Scatter plots with smoothed density showing the correlation of DNA methylation levels at different genic regions with gene expression. The plots reveal the relationship of DNA methylation and expression in benign and malignant tumors.

Levels of gene expression can be regulated by multiple types of epigenetic control mechanisms that include but are not limited to DNA methylation, histone modification, transcription factor binding and higher-order chromosomal structure. To understand the relationship of DNA methylation and gene expression, genome-scale gene expression profiles in 12 meningioma samples (five benign, two atypical and five malignant) were measured using the Illumina HumanHT-12 v4 Expression BeadChip. All but one sample was previously subjected to the Illumina Infinium HM450 DNA methylation BeadChip for DNA methylation analysis (Table 1). The relationship of the mean DNA methylation levels with the mean expression values was explored for DNA methylation probes targeting different genic regions. Included in the analysis were malignant and benign meningiomas samples with both DNA methylation and expression data available. An inverse correlation of DNA methylation with gene expression was observed for gene core promoter (TSS200) probes and 1^st^ exon probes (1stExon) with Spearman's rank correlation rho values of approximately −0.45, and less obvious for other categories of probes (Figure 3). However, the overlap of mean differential DNA methylation with differential expression between malignant and benign groups was not observed (Figure S2). Considering the sample size in our study is relatively small (see Methods), we included the publicly available array-based expression data of six malignant meningiomas and 43 benign meningiomas [7] to further explore potential correlation of DNA methylation with gene expression. Shown in Figure S3, we observed an inverse correlation of promoter (TSS200) DNA methylation with gene expression for both malignant and benign meningiomas with approximate rho values of −0.38; however, we did not see any overlap of differential DNA methylation with differential gene expression between malignant and benign tumors. Thus in general, the change of DNA methylation is not strongly associated with alteration of gene expression during malignant transformation of meningiomas.

We further investigated the 15 most hypomethylated genes and asked whether these genes have altered expression in malignant tumors. Most of these genes were lowly expressed in both benign and malignant meningiomas (Table S1); however, five genes (*ADCY3*, *GAS7*, *LAG3*, *LRR32* and *SPON2*) showed a trend of elevated expression (>3 fold in mean values) in malignant meningiomas. However, statistical significance was not detected (unpaired two-tailed t-test). Considering the sample size in our study is relatively small, we further assessed expression of these genes in the publicly available expression datasets mentioned above, but still did not observe statistical significance.

### DNA hypermethylation of CpG islands and the polycomb repressive complex

In addition to global DNA hypomethylation, focal DNA hypermethylation at CpG islands is also typical in human cancers [21], [29], [30] and is believed to contribute to the reduction of gene expression, especially at tumor suppressor genes. Analysis of gene expression data in the malignant and benign groups identified 164 differentially expressed genes (FDR = 1%), all of which were suppressed in the malignant group. This observation raised the question of whether gene suppression correlates with DNA hypermethylation of CpG islands in malignant meningiomas.

**Figure 4:**
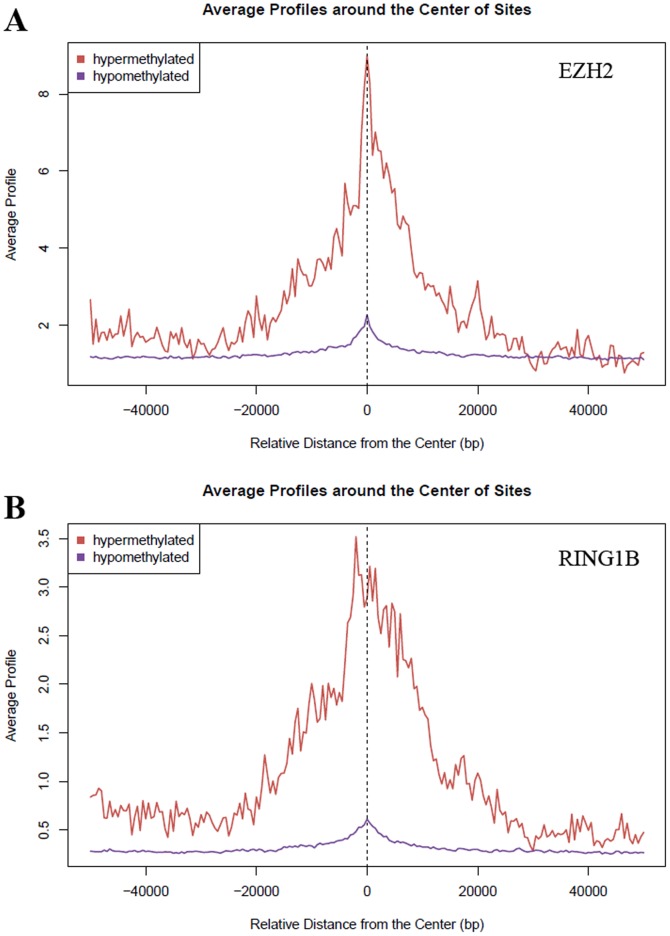
Enrichment of PRC proteins at hypermethylated CpG islands. Aggregation plots of EZH2 (*upper panel*) and RING1B (*lower panel*) ChIP-Seq signal intensities around the center of hypermethylated and hypomthylated CpG islands generated using Sitepro tool in Cistrome Analysis pipeline [42]. Hypermethylated CpG islands in malignant meningiomas are strongly correlated with the binding sites of polycomb repressive complex.

To explore DNA hypermethylation of CpG islands, CpG island probes with increased mean β values in malignant meningiomas compared to benign tumors (total 56,677) were chosen for statistical analysis to identify significantly hypermethylated CpG islands. The probes with decreased mean β values in malignant meningiomas (total 85,342) were also tested to identify significantly hypomethylated CpG islands. Significance Analysis of Microarray (SAM) analysis (FDR = 1%, see Methods) [31] identified 100 hypermethylated CpG islands and 4,449 hypomethylated CpG islands in malignant meningiomas, and we observed enrichment of hypermethylated CpG islands at coding exon regions and hypomethylated CpG islands at intron regions, accounting for 27.1% and 31.5% of identified CpG islands respectively (Table S2). When exploring the association of hypermethylated or hypomethylated CpG islands with binding sites of polycomb repressive complexes (PRC) in human embryonic stem cells, we found strong association of hypermethylated not hypomethylated CpG islands with binding sites of both the PRC1 protein RING1B and the PRC2 protein EZH2 with hypermethylated CpG islands. In contrast, hypomethylated CpG islands are not co-localized with PRC binding (Figure 4). Among 26 genes having hypermethylated CpG islands within close proximity of their transcription start sites (1 kb upstream and downstream), seventeen of them (65.4%) are PRC-target genes in early developmental stages and are likely to be also targeted by DNA methyltransferases in malignant meningiomas (Table S3). Expression of DNA methyltransferase such as *DNMT1* gene is elevated in malignant meningiomas (Figure S5). It is conceivable that DNMT1 is recruited by PRC component protein such as EZH2 [32] to induce DNA hypermethylation in malignant meningiomas. Within the PCR-targeting genes, DNA hypermethylation of *HOXA6* and *HOXA9* has also been implicated in recurrent meningiomas according to another study [25]. Examination of gene expression levels in 15 PRC-target genes with expression data available reveals a relatively low mean expression values in the benign group, most of which were also suppressed in the malignant group (Table S3). This is consistent with previous observations in other cancer types [33], [34], where CpG island containing genes temporarily silenced (poised) by PRC in normal tissues may acquire DNA methylation during cancer formation, resulting in permanent silencing.

**Figure 5:**
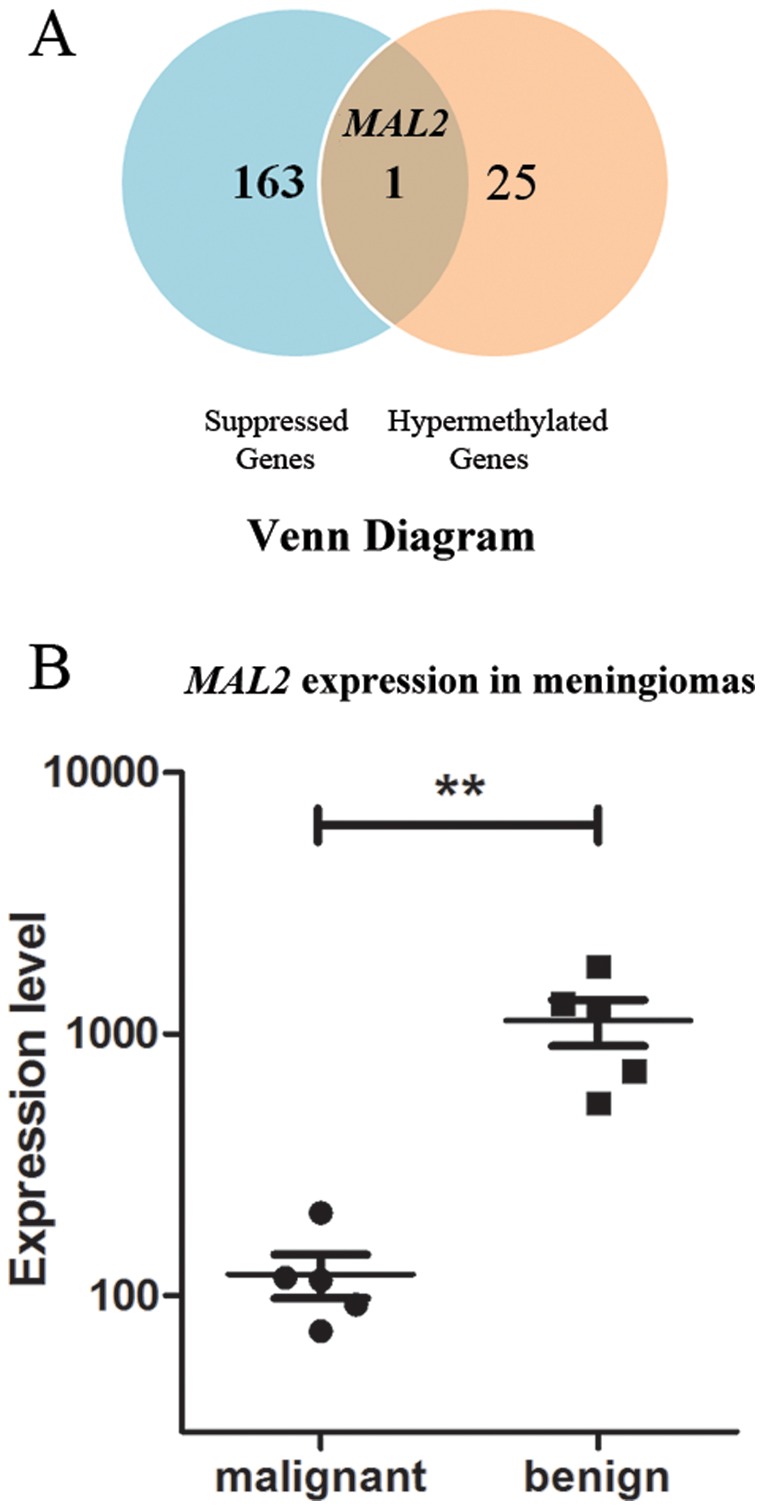
Gene *MAL2* hypermethylated and differentially expressed. A), Venn diagram showing overlap of suppressed genes with hypermethylated genes; B), Plot showing *MAL2* gene expression in malignant and benign meningiomas. *MAL2* is highly expressed in benign meningiomas, but is silenced in malignant meningiomas.

Examination of expression data of genes with hypermethylated promoter CpG islands also identified one gene, *MAL2*, with high expression levels in the benign meningiomas but low expression in malignant tumors (Table S3). Interesting to note, *MAL2* is the only overlapping gene that displayed suppression of gene expression and hypermethylation of a CpG island at its promoter region (Figure 5A). Expression of *MAL2* is significantly suppressed in the malignant sample group (*P* = 0.0022, two-tailed unpaired t-test, Figure 5B). The *MAL2* gene encodes a multispan transmembrane protein belonging to the MAL proteolipid family with function in transcytosis. To further support our analysis, we examined a publicly available gene expression array data for meningiomas [7], and found significantly suppressed *MAL2* gene expression in recurrent malignant samples compared to benign samples (*P* = 0.0096, two-tailed unpaired t-test). Thus, suppression of *MAL2* gene expression is tightly associated with malignancy of meningiomas.

**Figure 6:**
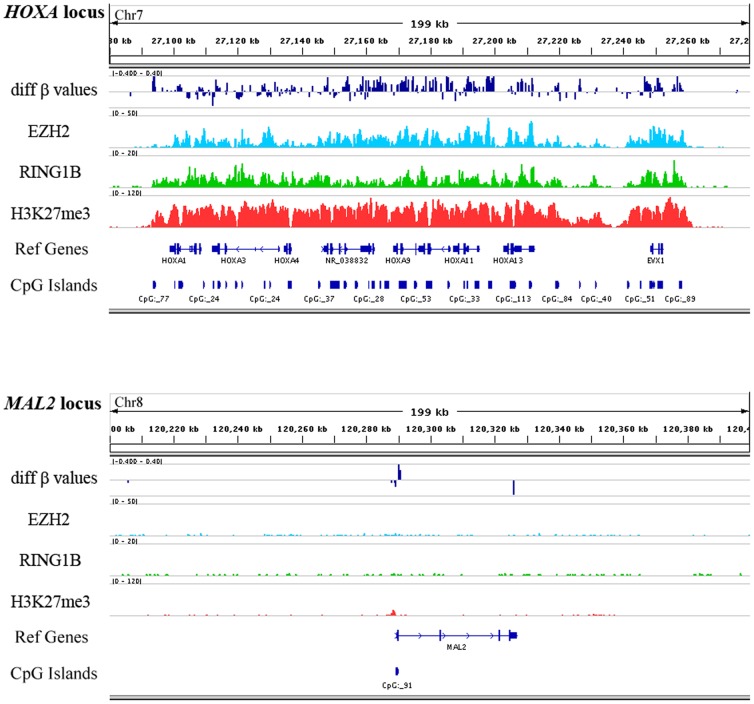
Comparison of epigenetic marks at *HOXA* and *MAL2* gene loci. CpG island DNA hypermethylation of *HOXA* and *MAL2* gene loci were visualized using the Integrative Genomics Viewer (IGV) together with tracks of ChIP-Seq profiles of EZH2, RING1B, H3K27me3 in human ES cells and tracks of reference genes and CpG islands. For the *HOXA* locus, CpG islands are hypermethylated in malignant meningiomas and co-localized with repressing EZH2, RING1B and H3K27me3 markers. In contrast, CpG island at the *MAL2* promoter is absent of those repressing markers.

DNA hypermethylation is generally associated with epigenetic switching from transient suppression by PRC to permanent suppression by DNA methyltransferases. In malignant meningiomas, the *HOXA* gene locus “poised” by PRC1 and PRC2 targeting was likely to undergo epigenetic switching [34], gaining DNA methylation (Figure 6) for permanent gene silencing. On the other hand, *MAL2* hypermethylation represents an example of *de novo* DNA methylation related suppression of genes, which are not targeted by PRC proteins and are actively transcribed in normal tissue.

## Discussion

To the best of our knowledge, the current study represents the first to interrogate whole-genome DNA methylation levels across three subtypes of meningiomas, and provides novel insights into the molecular pathophysiology of malignant transformation in meningioma. Globally decreased DNA methylation levels observed in the atypical and malignant meningiomas compared to their benign counterparts were in line with aggressiveness of this tumor type, and DNA hypomethylation was not restricted to specific gene regions. Malignant meningioma could be distinguished from atypical and benign ones according to DNA methylation patterns, suggesting the potential application of genome-wide DNA methylation profiling in specifying the diagnostic subtype of meningioma.

In our study, DNA methylation and gene transcription profiles were assessed in a group of patients with malignant, atypical, or benign meningiomas. Biological pathway analysis identified DNA hypomethylation events associated with the glioma pathway in malignant meningiomas. However, expression levels of the identified DNA hypomethylated genes was not significantly altered in the malignant group, suggesting change of DNA methylation does not directly contribute to altered gene expression. Consistent with that, we did not observe overlapping genes of differential DNA methylation at any genic regions with differential gene expression between malignant and benign meningiomas. In addition, we analyzed a publicly available dataset of glioma samples from the TCGA Data Portal. From a chosen group of twenty-seven glioma samples with both DNA methylation data and expression data available, we explored correlation of DNA methylation levels at different genic regions with gene expression. Similar to our findings in meningiomas, we did not observe strong correlation in the glioma dataset (Figure S4).

With genome-scale DNA methylation data, we also explored focal DNA hypermethylation in malignant meningiomas. Malignant tumors often demonstrate DNA hypermethylation at CpG island regions when compared to normal tissue [29], [30]. Prior studies have demonstrated hypermethylation at hundreds of CpG islands in astrocytomas, another common brain tumor [35]. Although the principle of focal hypermethylation has been well received when comparing cancer *versus* normal tissue, few studies have assessed differences in focal hypermethylation between malignant and benign tumors. For the malignant meningiomas in our study, 26 genes were identified as significantly hypermethylated at promoter CpG islands, including eight genes involved in the biological pathway of neurogenesis (*BARHL2*, *TLX3*, *FOXR1*, *HOXA11*, *HOXA6*, *HOXA9*, *OTX2* and *PAX3*). Interestingly, hypermethylated CpG islands in malignant meningiomas coincided with binding of polycomb repressive complexes (PRCs) in early developmental stages, whereas hypomethylated CpG islands did not show such a pattern. It is conceivable that epigenetic switching from poised gene silencing regulated by PRCs to permanent silencing by DNA methylation, which has been observed in the PC3 prostate cancer cell lines [34], also occurs in malignant meningiomas. Approximately 65% of the genes with DNA hypermethylation at promoter CpG islands were associated with PRCs, and the majority of them (87%) were suppressed in both benign and malignant meningiomas. This suggests the possibility of epigenetic switching on gene silencing. Previous studies have demonstrated that addition of methyl groups to cytosine is catalyzed by DNA methyltransferases (DNMTs), including DNMT1, DNMT3a, and DNMT3b in mammalian cells [36], [37], [38]. Increased expression of the DNA methytransferase *DNMT1* gene in malignant meningiomas (Figure S5) suggests that DNMT1 may be involved in maintaining focal DNA hypermethylation, as other studies have reported in cancer cells [39], [40]. DNMT1 has been previously shown to interact with PRC component EZH2 and to methylate PRC targeted genomic regions [32], therefore increased DNMT1 levels in malignant meningiomas may be recruited to PRC-targeted CpG islands and induce focal DNA hypermethylation. However, hypomethylated CpG sites are most likely not associated with PRC binding, therefore hypomethylated sites are unlikely to be affected by altered DNMT1 protein DNA levels. It is possible that global hypomethylation in malignant meningiomas results from disregulated DNA replication in the cell cycle.

The majority of genes hypermethylated at promoter CpG islands are suppressed in both malignant and benign meningiomas. We did, however, identify one exception, the *MAL2* gene. The *MAL2* gene product is an integral membrane protein with a functional role in lipid raft-mediated trafficking. A previous study in mouse esophageal carcinoma showed that loss of MAL expression is correlated to tumor progression, and ectopic expression of MAL prevents tumor formation by triggering apoptosis of tumor cells [41]. In our study, DNA hypermethylation of the *MAL2* gene was associated with a statistically significant reduction in gene expression. This might represent *de novo* gene silencing induced by DNA methylation in malignant meningiomas, and is potentially a good candidate biomarker (tumor suppressor) for further diagnostics.

The sample size in our study is relatively small, thus we are limited by statistical power from the analysis. For example, within identified DNA hypermethylated genes, only *MAL2* shows high statistical power (>99%) when expression data was compared between malignant and benign groups (Table S3). Theoretically, with increased sample size, statistical power will increase accordingly. For example, gene *PIGY3* shows 20% reduction in mean expression in malignant group. However, the statistical power is only 20.8%. If the sample size were doubled (10 samples for each group), the power would have increased to 50% (assuming the same mean values and standard deviation).

In summary, we provided a genome-scale map of DNA methylation in malignant, atypical and benign meningiomas. We further discussed the functional relevance of DNA methylation in tumor malignancy. Genes harboring significantly different methylation patterns between malignant and benign tumors offer a promising avenue for diagnostic utility. However, these findings require further assessment and validation prior to potential clinical application.

## Supporting Information

Figure S1 DNA methylation levels of glioma-related genes in meningiomas. Heatmap illustrating DNA methylation β values of core enriched genes in glioma-related biological pathways. Genes that belong to the core enrichment group were highlighted. The color in each cell represents gene DNA methylation levels (red: higher methylation; blue: lower methylation).
(TIF)

Figure S2 Differential DNA methylation *vs.* differential expression in meningiomas. The scatter plots with smoothed density for visualizing the relationship of differential DNA methylation with differential gene expression in our study. DNA methylation levels at core promoters (TSS200), remote promoters (TSS1500), 1^st^ Exons (1stEXON), 5′UTR, 3′UTR and gene body (Body) were separately correlated to gene expression using Spearman's test. The correlation coefficient value (rho) was included in the upper right corner of each plot.
(TIF)

Figure S3 DNA methylation *vs.* gene expression using publicly available expression data in meningiomas. The scatter plots with smoothed density for visualizing the relationship of promoter DNA methylation with publicly available gene expression data (see methods) in malignant and benign meningiomas (Figure S3A, S3B). The relationship of promoter differential methylation with differential gene expression was shown in Figure S3C.
(TIF)

Figure S4 DNA methylation *vs.* gene expression using publicly available data in glioma. The scatter plots with smoothed density for visualizing the relationship of glioma DNA methylation levels at different genic regions with gene expression data. Group mean values of twenty-seven glioma samples were used for analysis.
(TIF)

Figure S5 Expression levels of DNMT1 gene in meningiomas. A) Comparison of *DNMT1* gene expression in malignant and benign tumor samples in our study (five benign and five malignant samples)^+^. The data showed a trend of increased DNMT1 expression in malignant meningiomas, although statistically not significant; B) Comparison of *DNMT1* gene expression in malignant and benign tumors in the GEO database (forty-three benign and six malignant samples, see methods)*. Statistically significant increase of *DNMT1* gene expression in malignant meningiomas was observed (p<0.01, two-tailed *t*-test).
(TIF)

Table S1 Expression data of genes hypomethylated at core promoter regions. Expression levels for genes severely hypomethylated at core promoters were compared in malignant and benign meningiomas. Group mean and standard error of mean (SEM) values were included in the table.
(DOCX)

Table S2 Distribution of the CpG islands at different genomic regions. Distribution of the CpG islands at different genomic regions. Distribution at genomic regions such as gene promoter, gene downstream, 5′UTR, 3′UTR, coding exon, intron and distal intergenic regions was compared for the hypomethylated, hypermethylated and all CpG islands.
(DOCX)

Table S3 Expression of genes with hypermethylated CpG islands. Expression of genes with hypermethylated CpG islands at promoter regions. Group mean expression values for those genes were calculated for malignant and benign meningiomas. Also the information of PRC targeting was included in the last column.
(DOCX)
