# Supplementary figures and images for: DNA Methylation in the Malignant Transformation of Meningiomas
Source: PLoS One. 2013 Jan 22;8(1):e54114. doi: 10.1371/journal.pone.0054114 (PMC3551961; doi:10.1371/journal.pone.0054114)

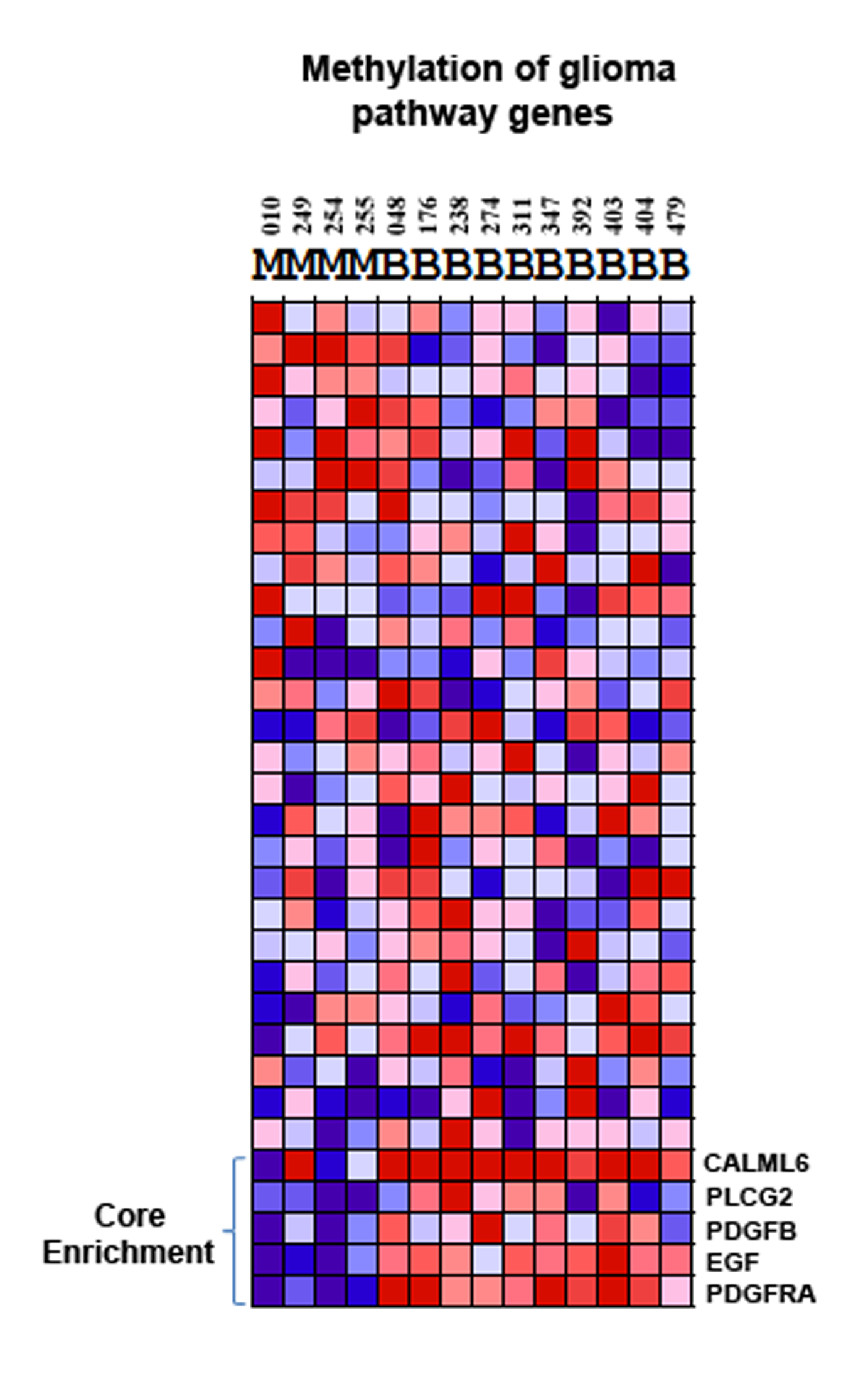

Supplement: Figure S1 — DNA methylation levels of glioma-related genes in meningiomas. Heatmap illustrating DNA methylation β values of core enriched genes in glioma-related biological pathways. Genes that belong to the core enrichment group were highlighted. The color in each cell represents gene DNA methylation levels (red: higher methylation; blue: lower methylation). (TIF) [file pone.0054114.s001.tif]

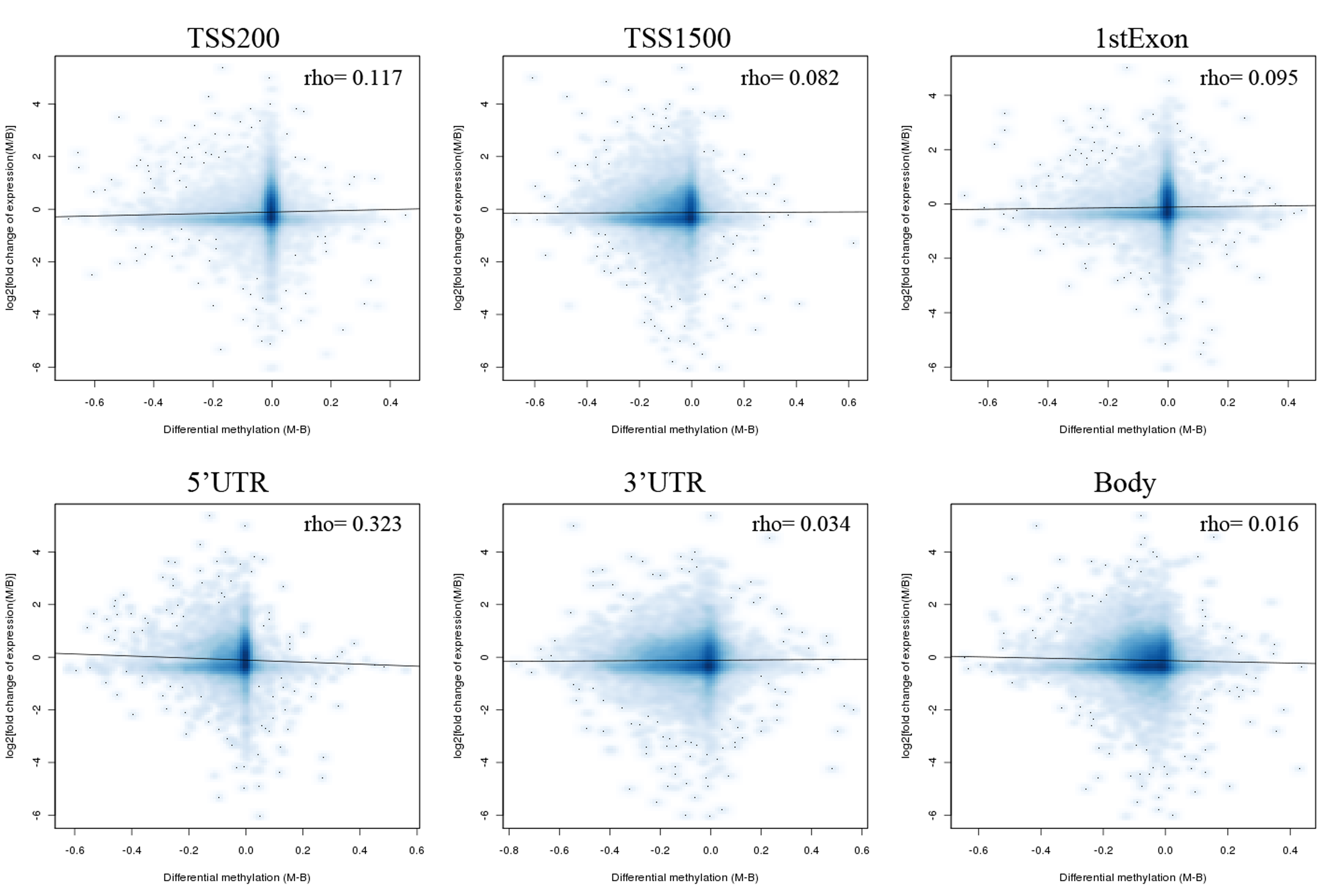

Supplement: Figure S2 — Differential DNA methylation vs. differential expression in meningiomas. The scatter plots with smoothed density for visualizing the relationship of differential DNA methylation with differential gene expression in our study. DNA methylation levels at core promoters (TSS200), remote promoters (TSS1500), 1st Exons (1stEXON), 5′UTR, 3′UTR and gene body (Body) were separately correlated to gene expression using Spearman's test. The correlation coefficient value (rho) was included in the upper right corner of each plot. (TIF) [file pone.0054114.s002.tif]

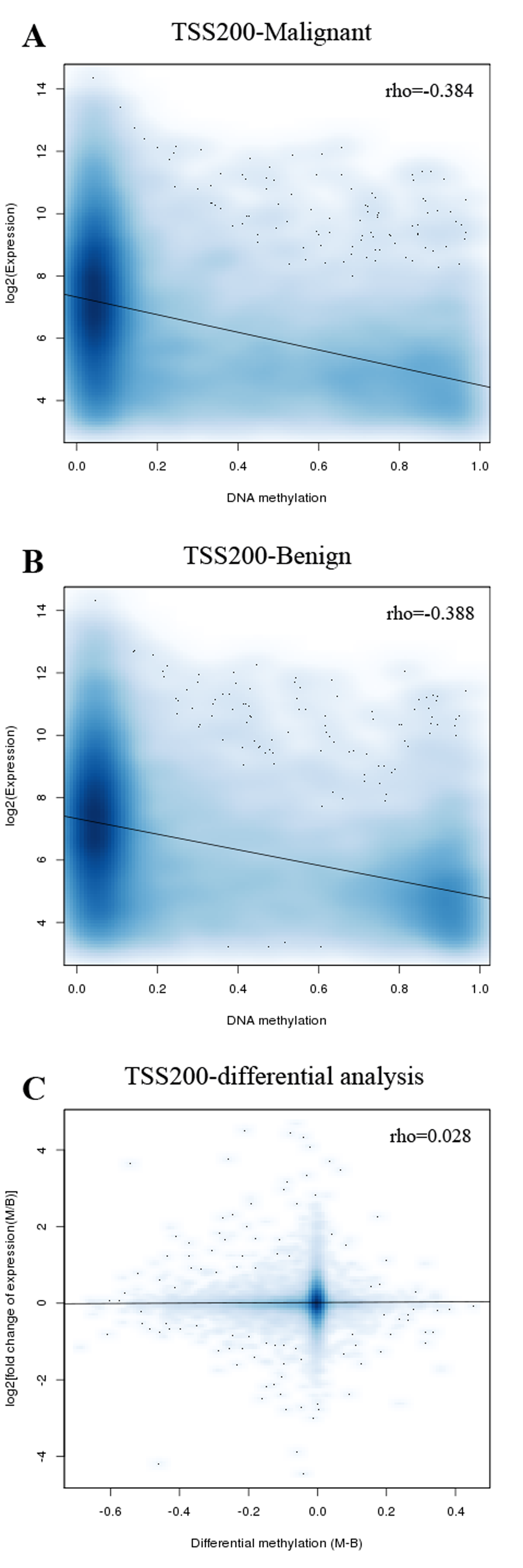

Supplement: Figure S3 — DNA methylation vs. gene expression using publicly available expression data in meningiomas. The scatter plots with smoothed density for visualizing the relationship of promoter DNA methylation with publicly available gene expression data (see methods) in malignant and benign meningiomas (Figure S3A, S3B). The relationship of promoter differential methylation with differential gene expression was shown in Figure S3C. (TIF) [file pone.0054114.s003.tif]

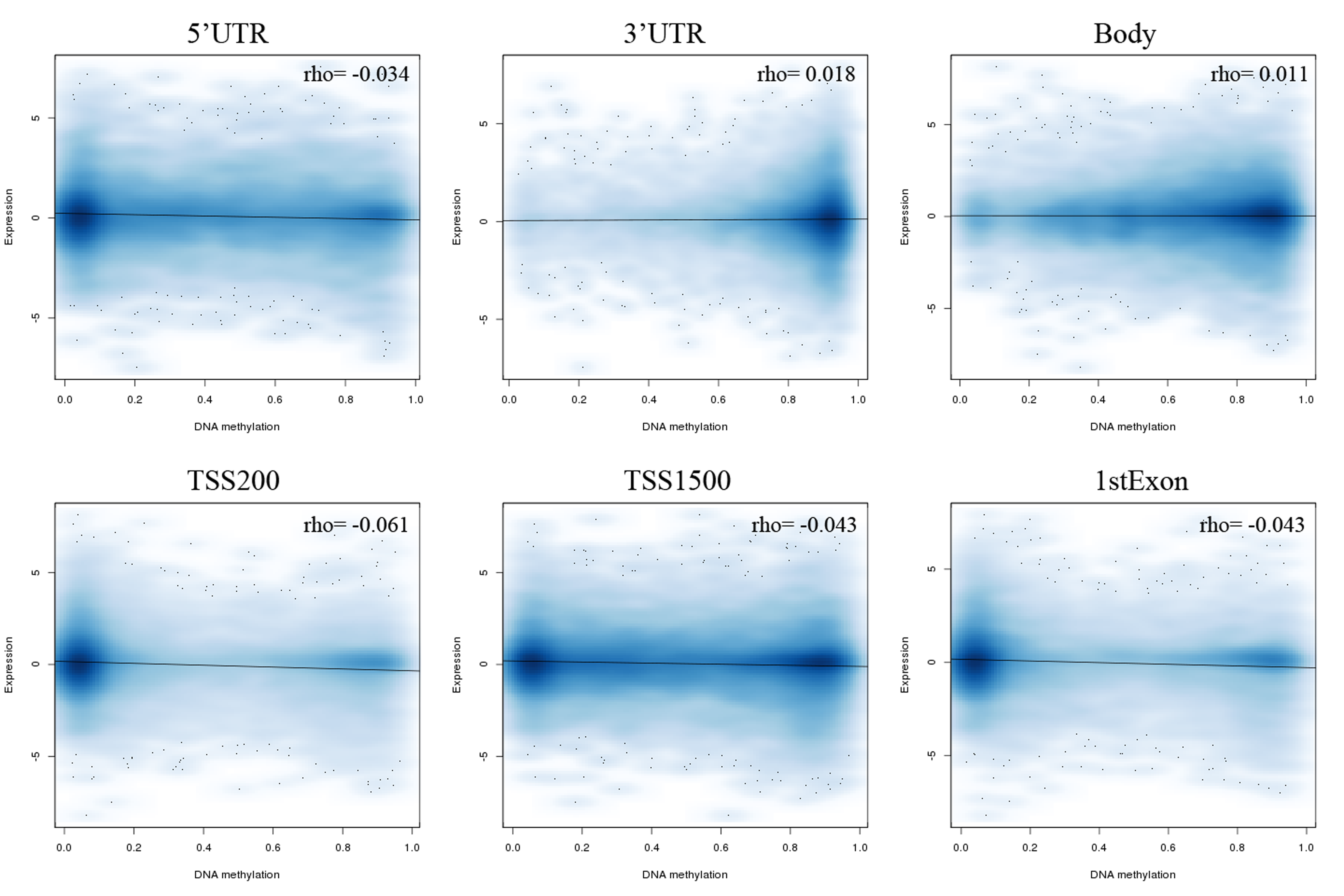

Supplement: Figure S4 — DNA methylation vs. gene expression using publicly available data in glioma. The scatter plots with smoothed density for visualizing the relationship of glioma DNA methylation levels at different genic regions with gene expression data. Group mean values of twenty-seven glioma samples were used for analysis. (TIF) [file pone.0054114.s004.tif]

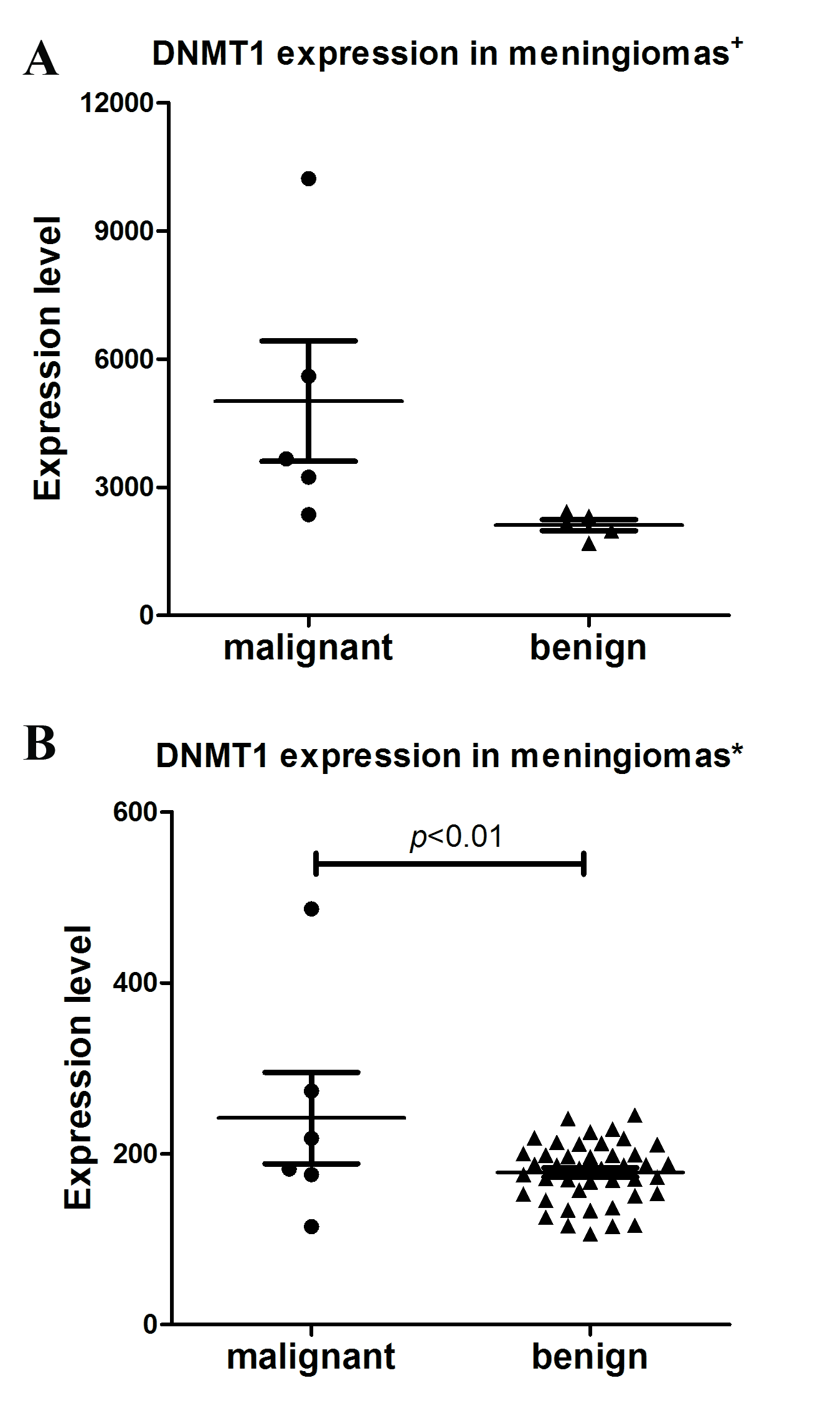

Supplement: Figure S5 — Expression levels of DNMT1 gene in meningiomas. A) Comparison of DNMT1 gene expression in malignant and benign tumor samples in our study (five benign and five malignant samples)+. The data showed a trend of increased DNMT1 expression in malignant meningiomas, although statistically not significant; B) Comparison of DNMT1 gene expression in malignant and benign tumors in the GEO database (forty-three benign and six malignant samples, see methods)*. Statistically significant increase of DNMT1 gene expression in malignant meningiomas was observed (p<0.01, two-tailed t-test). (TIF) [file pone.0054114.s005.tif]
