# Supplementary material for: DNA Methylation in the Malignant Transformation of Meningiomas
Source: PLoS One. 2013 Jan 22;8(1):e54114. doi: 10.1371/journal.pone.0054114 (PMC3551961; doi:10.1371/journal.pone.0054114)
Supplement: Table S1 — Expression data of genes hypomethylated at core promoter regions. Expression levels for genes severely hypomethylated at core promoters were compared in malignant and benign meningiomas. Group mean and standard error of mean (SEM) values were included in the table. (DOCX) [file pone.0054114.s006.docx]

**Table S1 Expression data of genes hypomethylated at core promoter regions***

| Gene ID | Mean Expression (malignant) | STD ^#^ (malignant) | Mean Expression (benign) | STD (benign) | Statistical power^$^ |
| --- | --- | --- | --- | --- | --- |
| *ADCY3* | 7078.00 | 5184.37 | 1783.10 | 595.82 | 62.1% |
| *ANGPTL1* | 101.68 | 26.46 | 112.25 | 19.28 | 11.2% |
| *C1orf113* | 106.89 | 24.38 | 146.26 | 20.02 | 79.7% |
| *C4BPA* | 101.17 | 48.48 | 100.15 | 14.03 | 5.0% |
| *C7orf65* | 131.99 | 15.68 | 149.28 | 19.38 | 34.1% |
| *DIAPH3* | 258.70 | 71.88 | 155.59 | 36.19 | 81.7% |
| *DLC1* | 222.92 | 261.24 | 134.51 | 37.49 | 11.6% |
| *GAS7* | 823.42 | 702.44 | 197.93 | 60.22 | 51.0% |
| *LAG3* | 1304.50 | 2141.89 | 141.43 | 42.57 | 22.9% |
| *LOC100130872* | 114.37 | 22.93 | 127.86 | 25.17 | 14.4% |
| *LRRC32* | 1812.00 | 2759.81 | 525.27 | 194.96 | 18.0% |
| *MAMDC2* | 556.99 | 769.76 | 214.45 | 77.56 | 16.8% |
| *MSLNL* | 112.08 | 19.76 | 127.50 | 16.11 | 27.2% |
| *SLC1A5* | 559.40 | 358.29 | 296.07 | 108.23 | 35.0% |
| *SPON2* | 539.70 | 759.96 | 133.55 | 38.78 | 22.3% |

*Hypomethylated gene promoters were selected based on the criteria defined in Methods.

^#^STD: standard deviation.

^$^Statistical power was calculated using the mean and STD values of malignant and benign groups (two-tailed test, alpha error level 5%).
