# Supplementary material for: DNA Methylation in the Malignant Transformation of Meningiomas
Source: PLoS One. 2013 Jan 22;8(1):e54114. doi: 10.1371/journal.pone.0054114 (PMC3551961; doi:10.1371/journal.pone.0054114)
Supplement: Table S2 — Distribution of the CpG islands at different genomic regions. Distribution of the CpG islands at different genomic regions. Distribution at genomic regions such as gene promoter, gene downstream, 5′UTR, 3′UTR, coding exon, intron and distal intergenic regions was compared for the hypomethylated, hypermethylated and all CpG islands. (DOCX) [file pone.0054114.s007.docx]

**Table S2 Distribution of the CpG islands at different genomic regions***

|  | All CpG islands in the methylation array | Hypermethyalted CpG islands | Hypomethylated CpG islands |
| --- | --- | --- | --- |
| Promoter (<=1000 bp) | 35.30% | 11.50% | 15.40% |
| Promoter (1000-2000 bp) | 2.00% | 3.10% | 2.30% |
| Promoter (2000-3000 bp) | 0.80% | 4.20% | 0.70% |
| Downstream (<=1000 bp) | 1.00% | 1.00% | 2.10% |
| Downstream (1000-2000 bp) | 0.80% | 0.00% | 1.70% |
| Downstream (2000-3000 bp) | 0.60% | 2.10% | 0.90% |
| 5'UTR | 12.40% | 5.20% | 4.30% |
| 3'UTR | 2.00% | 7.30% | 4.30% |
| Coding Exon | 12.60% | 27.10% | 20.70% |
| Intron | 20.80% | 15.60% | 31.5% |
| Distal Intergenic | 11.80% | 22.90% | 16.2% |

*Reference genes in the genome assembly hg18 were used to calculate the distribution of the CpG islands.
