# Supplementary material for: DNA Methylation in the Malignant Transformation of Meningiomas
Source: PLoS One. 2013 Jan 22;8(1):e54114. doi: 10.1371/journal.pone.0054114 (PMC3551961; doi:10.1371/journal.pone.0054114)
Supplement: Table S3 — Expression of genes with hypermethylated CpG islands. Expression of genes with hypermethylated CpG islands at promoter regions. Group mean expression values for those genes were calculated for malignant and benign meningiomas. Also the information of PRC targeting was included in the last column. (DOCX) [file pone.0054114.s008.docx]

**Table S3 Expression of genes with hypermethylated CpG islands**

| Gene ID | Expression (benign, mean) | STD | Expression (malignant, mean) | STD | PRC binding in ESC* | Statistical power^$^ |
| --- | --- | --- | --- | --- | --- | --- |
| *BARHL2* | 99.04 | 17.98 | 90.30 | 26.07 | Yes | 9.5% |
| *BOLL* | 124.20 | 26.61 | 98.27 | 20.96 | No | 40.2% |
| *CCDC140* | 122.00 | 15.88 | 108.92 | 28.63 | Yes | 14.5% |
| *CLDN23* | 158.55 | 32.05 | 239.68 | 105.13 | No | 37.9% |
| *COL9A1* | 110.78 | 17.00 | 153.19 | 75.38 | Yes | 23.3% |
| *FLJ32063* | 122.28 | 15.64 | 107.19 | 43.15 | Yes | 11.4% |
| *FOXR1* | 135.68 | 24.70 | 106.57 | 23.41 | No | 48.1% |
| *HCN2* | 124.70 | 16.70 | 131.03 | 81.43 | No | 5.3% |
| *HOXA11* | 88.11 | 10.85 | 75.96 | 19.62 | Yes | 22.8% |
| *HOXA11-AS1* | 118.91 | 19.04 | 108.59 | 31.01 | Yes | 9.7% |
| *HOXA6* | 179.28 | 23.13 | 175.79 | 35.16 | Yes | 5.4% |
| *HOXA9* | 103.25 | 10.77 | 88.14 | 16.03 | Yes | 41.7% |
| *HTR1B* | 128.16 | 30.62 | 89.81 | 13.90 | Yes | 72.2% |
| *LOC389493* | 117.58 | 18.81 | 95.36 | 23.52 | No | 37.8% |
| *LOC645323* | 125.51 | 14.81 | 468.33 | 669.22 | Yes | 20.9% |
| *MAL2* | 1123.46 | 502.92 | 120.44 | 51.35 | No | 99.3% |
| *MIR9-2* | N/A | N/A | N/A | N/A | Yes | N/A |
| *NKX2-2* | 126.78 | 10.80 | 454.62 | 785.92 | Yes | 15.4% |
| *NKX2-4* | N/A | N/A | N/A | N/A | Yes | N/A |
| *OTX2* | 102.16 | 13.05 | 82.25 | 19.69 | Yes | 47% |
| *PAPOLB* | 107.55 | 14.48 | 84.01 | 16.05 | No | 68.3% |
| *PAX3* | 112.49 | 14.77 | 93.35 | 23.81 | Yes | 33.3% |
| *PIGY* | 4153.67 | 754.99 | 3373.52 | 1324.35 | No | 20.8% |
| *PON3* | 136.09 | 31.54 | 141.89 | 69.66 | No | 5.3% |
| *TCF21* | 126.95 | 18.24 | 105.16 | 21.74 | Yes | 40.4% |
| *TLX3* | 124.63 | 22.20 | 112.31 | 20.30 | Yes | 15% |

*ChIP-seq binding profiles of polycomb-repressive complex (PRC) proteins EZH2, RING1B in human embryonic stem cells (ESC) were used to determine gene association with PRC.

^$^Statistical power was calculated using the mean and STD values of malignant and benign groups (two-tailed test, alpha error level 5%).
